# The role of supplier-induced demand on the occurrence of information overload in managerial reporting environments

**DOI:** 10.1371/journal.pone.0307671

**Published:** 2024-07-25

**Authors:** Peter Gordon Rötzel

**Affiliations:** Faculty of Engineering and Computer Sciences, University of Applied Sciences Aschaffenburg, Aschaffenburg, Germany; Istanbul Technical University: Istanbul Teknik Universitesi, TÜRKIYE

## Abstract

This article develops a model showing how information reporters influence information load among decision makers and generate supplier-induced information demand (SID). The intra-corporate information-providing process is an expert market with information asymmetry. I show that information overload occurs as an SID and is the result of informational overconsumption deliberately caused by the supplying reporter. My analysis highlights that the information overload depends on the specificity of information. It also shows that the decision maker may face a hold-up situation in light of switching costs. The more specific the information needed, the higher the threat of information overload. The strategic content of information tempts reporting managers to overload the decision maker for the purpose of increasing their reporting transfer price and to discourage the decision maker from getting the information from another reporting manager. Although the decision maker knows a part of the information demand, information overload involves the cost of using unnecessary inputs, information overload occurs as an SID of information, even if other competing reporting managers exist. My analysis demonstrates that information overload can occur due to uncertainty and opportunism of both the decision maker and reporting managers.

## Introduction

Decision makers need information in order to choose optimally among alternatives; such information is often provided by managerial reporting. Intra-corporate information suppliers not only provide the information necessary for decision makers, but they can also act as experts in determining the decision makers’ requirements. Because of information asymmetry, decision makers do not know exactly how much information or managerial reporting is optimal. Typically, the decision maker first gets a recommendation from the information supplier in terms of the set of information needed. After finally receiving the information, most decision makers do not determine the veracity of the prior recommendation. In such situations, managerial reporting can be handled as a credence good, and the reporting process can be seen as an expert market e.g. [1].

Research has found that in expert markets with credence goods, suppliers take advantage of information asymmetry because they have an incentive to cheat on services. This phenomenon occurs in expert markets like health [2–5] or law [6, 7]. If it will increase their benefits, information suppliers tend to provide more information than necessary. Thus, decision makers with ex ante uncertainty about the set of information needed to make an optimal decision could be overloaded precisely because they want to reduce their uncertainty by inquiring with a reporting manager.

Studies to date have provided clear evidence that increased amounts of relevant information up to a certain level lead to better decision making [8–14]. Accounting research has found, however, that problems may arise from exceeding that certain threshold. The resulting effect is information overload (e.g. [15]). A twofold problem then arises: on the one hand, the decision maker has to decide with uncertainty because information underload, which can be reduced by inquiring with an expert in the form of a reporting manager. On the other hand, that step to reduce uncertainty and to increase the set of information could lead to an information overload if the reporting manager induces a demand for information beyond the optimum. Consequently, the problem of self-induced information overload could be extended by supplier-induced information (SID) overload.

Concerning information load, decision makers’ uncertainty problem is two-sided: first, the decision maker is uncertain about the set of information needed. Research has found that decision makers tend to gather more information than necessary, causing a self-imposed information overload [16]. Several studies show that decision making performance decreases in such cases when decision makers are overconfident in their ability to handle the set of information requested [13, 14, 17–20]. Second, the decision maker obtains reports from subordinate reporting managers, who themselves are uncertain about the decision maker’s information demand. An experimental study by Chervany & Dickson [21] showed that decision makers who used summarized data like management reports (1) made decisions of higher quality, (2) needed less time to make the decision, and (3) had more confidence than decision makers using disaggregated data. On the downside, several studies have shown that the decision maker’s subordinates, particularly in major corporations, tend to report more information than necessary. Organizational changes can lead to either greater information processing requirements (IPRs) owing to increasing communication and coordination [22, 23] or to lower IPRs through corporate standards, procedures, or rules [23–25]. In other words, there is a correlation between corporate size and the set of information the decision maker receives through reporting [16]. Therefore, the organizational component is an additional important factor influencing the occurrence of information overload [24, 26, 27].

In a simple case, managerial reporting consists of one decision maker who receives the report and one reporter who provides the information. Before receiving the managerial report, the decision maker consults the reporter to determine the set of information he needs. It is assumed that there is no second option and that within the company, only a single reporter can provide the specific information needed. Such a monopsony enables us to focus on the incentive issues involved without neglecting the strategic competition considerations. To illustrate, suppose our single decision maker has to make an investment decision, which generates a certain demand for information. The decision maker consults the reporting expert, who gets transfer prices for either consulting or reporting. Both the decision maker and the reporter are on the payroll of the same company. If information asymmetry is not present, the company’s benefit is greatest when the reporter is honest and provides the set of information that will lead to the maximal decision making performance possible. If the reporter’s gain is not congruent with corporate performance but is affected by advantages of reporting—such as reputation, status, or transfer pricing—the reporter could be tempted to provide more information than necessary.

In a case with one decision maker and various reporters, the decision maker may inquire with another reporter to determine the information demand and to reduce the uncertainty. There is theoretical and empirical evidence that in markets having an ex ante uncertainty about the optimal amount of a good (in this example, the information relevant for decision making) combined with an information asymmetry on the part of the supplier, the suppliers tend to oversupply consumers.

Despite the crucial rules of both information overload caused by reporting managers in management reporting and the decision maker’s two-sided uncertainty concerning information demand these aspects have yet been to be examined in the literature. Studies to date have examined the decision maker as the entity who determines the information load. Accordingly, the reporting behavior of subordinate managers and their influence on the set of information reported to decision makers in terms of induced demand have not yet been examined. If subordinates are aware of both the decision maker’s information overload risk and the consequences of decreasing decision making performance, they might take advantage and maximize their self-interest.

This research has three objectives: First, it examines the influence of supplier-induced demand in terms of information load on the decision-making performance by using game theory. Second, it analyzes the effects of information overload resulting from inefficient reporting behavior. Third, it examines the additional problem of whether and how the decision maker may be affected by overconfidence.

My article presents a model that is relevant to the information load and decision science literature. In studies to date, the systematic threat of information overload has not yet been analyzed in terms of supplier-induced demand. Deliberate information overload in terms of information asymmetry situation has not yet been examined in the literature.

My results have important implications for designing incentives for management reporting in a superior-subordinate relationship. In particular, the model shows, first, that information overload can occur as a supplier-induced demand of information, even if other competing reporting managers exist. This finding may offer a structural basis for applied research on reporting manager-induced information demand. Moreover, the provided model may explain why briefing efforts are often greater than briefing benefits. As in most models with switching costs, reporting managers are assumed as wishing to draw the decision maker’s attention in the first period. My analysis demonstrates that information overload can occur because of the uncertainty and opportunism of both decision maker and reporting managers.

The remainder of this article is organized as follows. Section 2 sets out the basic aspects of information load and supplier-induced information demand theory. Section 3 describes the model, which combines information load and supplier-induced information demand, whereas section 4 analyzes the resulting equilibria. Section 5 discusses the relaxations of assumptions, presents the results, and evaluates their managerial implications. Section 6 discusses the results. Finally, section 7 presents the conclusions.

## Information load and supplier-induced information demand in decision making processes

If the set of information provided by the reporting manager exceeds the decision maker’s information processing capacity, then information overload occurs [28–30]. The processing capacity of a decision maker is fairly limited by a phenomenon called bounded rationality [31] as well as general capacity [28]. Research on information overload relating to management decisions has shown an interrelationship between information load and decision making performance in the areas of accounting [13, 14, 25, 32–35] or management information systems (MIS) [36]. Other studies have examined information overload in the context of other research areas like organization science [24, 26] or marketing and consumer research [37–39].

**Fig 1:**
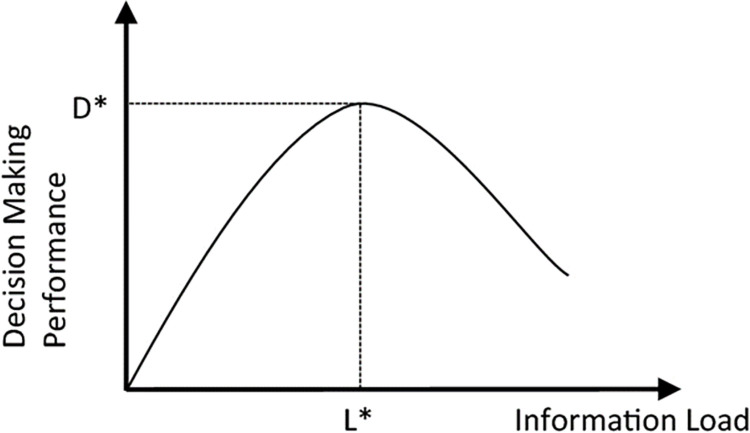
Interrelationship between set of information and decision making performance (Roetzel 2019).

The theoretical basis of information overload is borrowed from cognitive psychology, such as Simon and Newell [40], Schroder et al. [29], and Miller [28]. Here, the basic characteristics of the human information processing system are serial processing, small short-term memory, and an infinite long-term memory. The relation between information load and decision making performance can be modeled as an inverted-U curve [29, 41–44] (see Fig 1). In that model, a decision maker is defined as a system that processes information inputs into decision outputs. The decision maker’s capability to integrate information into decisions is limited and is thought to follow a bell-shaped curve. If the provided information increases, the decision maker’s set of information, which is integrated into the decision output, initially rises. But beyond some point, further increases in the set of information provided leads to a decrease in the set of information integrated into decision outputs. Chewning & Hanell [13] showed in experiments that further information provided beyond this point rapidly reduces decision-making performance. Beyond that point, the decision maker’s output reflects a lesser utilization of the available information. O’Reilly [27] argued that the subsequent information overload results from the inability of managers to integrate the additionally provided information in the decision-making process. It is assumed that the individual decision maker has experienced information overload at the top point (L*/D*) of the inverted U-curve, where the set of information integrated into the decision output begins to decline [29, 41–44]. If the reporting manager provides information at an amount greater than L*, overload occurs. This kind of informational overconsumption is discussed in literature, specifically the second-degree price discrimination literature [45].

Concerning the problem of information load and assuming information asymmetry, the theory of supplier-induced demand suggests that a supplier might use superior information to tempt a consumer to demand a greater quantity of a good than necessary in terms of a Pareto efficient level, which results in a welfare loss. In terms of the decision making process, the SID effect may appear when information asymmetry exists between a decision maker (consumer) and a reporting manager (supplier), who provides a report as a bundle of information (good). The information demand is caused by the necessity to make a decision (e.g., an investment decision) for which detailed information from the responsible reporting manager is necessary. However, if the decision maker is affected by information overload, the decision is suboptimal at the corporate level (welfare loss).

On the one hand, from the decision maker’s view, the set of information needed is only partially known. On the other hand, from the reporting manager’s view, detailed information about the decision-making basis is available, but the reporting manager is uncertain about the exact set of information required by the decision maker ex ante. It is assumed that all information is relevant, but that there is a decreasing marginal usage of information on the decision maker’s side.

The decision maker faces the problem of making a decision based on management accounting information, but only the reporting manager can determine the set of information that is needed for making this decision. The report provided by the reporting manager contains the set of information the decision maker orders. Before the reporting manager provides a report, there is a briefing in which the decision maker exhibits the information demand. Afterward, the decision maker may order the subordinate reporting manager to compile a management accounting report of two sizes (small report, large report). The decision maker always has the opportunity to choose another reporting manager. It is assumed that there exist economies of scope between the briefing and the reporting. The briefing is not free for the decision maker, who at least spends some coordination time with the reporting manager. This cost denotes a hold-up problem [46], where after the briefing, the reporting manager may increase the set of information to enhance the reporting transfer price without fearing that the decision maker might reject the offer because of switching costs. Therefore, an information overload occurs whenever the reporting manager deliberately overloads the decision maker in order to get a higher reporting transfer price by providing a large report, even though a small report would suffice. Consequently, three results may occur for the decision maker: (1) the report matches the information demand (minor demand, small report provided; major demand, large report provided); (2) the decision maker faces information overload (minor demand, large report provided); and (3) the decision maker faces information underload (major demand, small report provided).

The following model deals with the question of how SID, in terms of information load, influences the decision-making performance.

## Model

There is a decision maker *D* with a set of *K* decisions to make, *d*_*k*∈{1,2,…,*K*}_, and a set of *N* subordinate reporting managers, *R*_*i*∈{1,2,…,*N*}_. First, the decision maker *D* has to make a decision and demand detailed information from the reporting manager. The information demand might be minor (state *γ*, with a probability *μ*∈[0,1]) or major (state Γ, with a probability 1−*μ*). That set of information is defined as *x*_*d*_. Contrary to the decision maker *D*, the reporting manager (*R*_*i*_) can differentiate between *γ* and Γ. The decision maker and the reporting manager are facing costs for briefing at the level of *b*≥0. Ex post, the reporting manager can satisfy the decision maker’s information demand with an effort of *ε*_*γ*_≥0 if the state is *γ*, and *ε*_Γ_>*ε*_*γ*_ in turn. The reporting manager provides the set of information *x*_*s*_ in the report. The transfer price to the reporting manager is an internal compensation paid by the decision maker (i.e., management visibility, reputation). From the decision maker’s view, the expected cost of provided information (IC) is


$$IC=b+\varepsilon_{\gamma }\mu +\varepsilon_{\Gamma }(1-\mu ).$$ (1)

As shown, the reporting manager needs briefing to assess the information demand of the decision maker. It is assumed that the reporting manager knows the decision maker’s information demand after the briefing. This implies that after briefing, there might be a one-sided uncertainty only concerning the set of information that has to be provided by the reporting manager. This briefing is necessary to provide the report, which leads to some economies of scope between the briefing and the reporting. These economies of scope are likewise switching costs and measured by *b*. The exact level of the decision maker’s information demand is not determinable, and the information demand consists of verifiable and subjective parts. Moreover, the information could be interpreted as a credence good [5]. Furthermore, it is assumed that there are observable characteristic inputs for large reports, which are denoted by Θ *ϵ* [0, *ε*_Γ_−*ε*_*γ*_]. The key effect of that assumption is that decision makers can reject a report when they think they might get a better report from another reporting manager. The decision maker’s ability to evaluate the inputs, which are only used in large reports, causes the reporting manager to incur a negative transfer price at the level of Θ (e.g., in terms of negative management visibility). Therefore, the reporting manager might be tempted to recommend a large report when a small report would be adequate due to the resulting higher transfer price. Consequently, Θ denotes the cost of overloading the decision maker with too much information. In turn, the positive cost of overloading could be interpreted as the reporting manager’s penalty when he is caught overloading.

As mentioned, the decision maker pays a briefing transfer price E^*b*^ to the reporting manager. Therefore, the reporting managers may present two different transfer prices of reporting to the decision maker. These transfer prices depend on the use of the characteristic inputs. The reporting manager *R*_*i*_ determines a briefing transfer price $E_{i}^{b}\geq 0$ and a reporting transfer price $E_{i}^{*}$ if characteristic inputs are used (large report), and a reporting transfer price $E_{i}^{\prime }$ (small report) otherwise.

In the analysis, a dynamic game is used. In the first period of the game, the decision maker knows all of the transfer prices of reporting of all reporting managers. This assumption is needed to avoid the hold-up situation in the first period described above. To illustrate, suppose the decision maker already has experience in prior briefings with the reporting managers and can evaluate the values of their reporting. Due to the chain of command, reporting managers may not be able to turn down a report request of the decision maker, but they can work with minimum effort, which is linked with negative reputation. It is assumed that in the case of an order for information, the state is denoted by $E_{i}^{\prime }>\varepsilon_{\gamma }$ and $E_{i}^{*}>\varepsilon_{\Gamma }$, so that the reporting manager does not face a loss. In turn, the decision maker could reject the offered report after briefing. In the second period, the report is provided by the reporting manager if the decision maker accepts the recommendation in the first period.

Research has found that many situations of incomplete information cannot be represented as static or strategic-form games [2, 47]. Therefore, a pure-strategy Perfect Bayesian Equilibria (PBE) is used. The decision maker’s strategy determines both which reporting manager to order and the order in which reporting managers should be consulted. Furthermore, the strategy specifies whether or not to use an offered report, depending on the combination of values in the game’s first stage. It is assumed that reporting managers cannot observe the decision maker’s past choices.

The reporting manager’s *R*_*i*_ strategy consists of a recommendation strategy (offer small report, offer large report) and a transfer price set ($E_{i}^{b},\;E_{i}^{\prime },\;E_{i}^{*}$). The recommendation strategy depends on both the decision maker’s observed information demand and the transfer price set. Truthful behavior can be observed when the reporting manager offers a small report if the decision maker’s information demand is minor Or a large report if the information demand is major.

The decision maker’s payoff is the transfer price of the information supply to make the decision, less the sum of all the transfer prices paid to reporting managers. The expected payoff of the decision maker is calculated using the initial belief *μ*. Moreover, the Bayesian updating rule is used every time the decision maker gets an offer. The reporting manager’s *R*_*i*_ payoff equals the difference between the reporting transfer prices and the efforts and is computed by using the distribution of information demand among open decisions.

It is assumed that decision makers accept a report if they are indifferent between rejecting and accepting. If opportunism is absent and reporting managers are indifferent between offering a large or a small report, they offer a small report. In such a case, therefore, deterministic recommendation and acceptance strategies are highlighted. It is assumed that there is at least one reporting manager who can provide the information with a positive probability in equilibrium. Furthermore, all reporting managers have the same transfer price set ($E_{i}^{b},\;E_{i}^{\prime },\;E_{i}^{*}$). Finally, it is assumed that there are at least two reporting managers.

Consequently, the analysis begins by imposing the following assumption, which takes account of the existence of competition among the reporting managers. They are able to react quickly if one of them tries to undercut the others.

**Assumption 1**: There are competing reporting managers who provide a small report at their effort level (zero benefit), but could not provide a large report. These reporting managers offer the transfer price set (*b*, *ε*_*γ*_, *ε*_*γ*_).

The theoretical basis of competing reporting managers provide small reports are opportunistic agents using their information base without a disadvantage (e.g., case workers or section managers) (e.g. [48, 49]).

**Assumption 2**: If the decision maker’s information demand is major, the first reporting manager inquired will provide a report if the decision maker accepts.

The idea behind assumption 2 is that a major information demand offers benefits above zero.

Before the briefing, the decision maker is uncertain about the information demand. It is assumed that the briefing enables the decision maker to realize the information demand, that is, that the decision maker might get that knowledge by interpreting the recommendation of the reporting manager or by the determination process in the briefing. It is assumed that the decision maker does not overestimate the information demand after briefing, and is therefore not affected by overconfidence. That leads to the following assumptions:

**Assumption 3**: The decision maker reduces the uncertainty about the information demand after the briefing.

**Assumption 4**: The decision maker uses only the necessary set of information to make a decision and is not affected by overconfidence.

An individual’s level of overconfidence can lead to information overload (e.g. [15]). Assumption 4 excludes the effect of overconfidence to simplify the model.

## Equilibria

In order to determine the possible equilibria, I developed a model based on two earlier studies by Wolinsky [2] and Alger and Salanié [47]. Wolinsky [2] used a model in which equilibria are either efficient or involve inefficient specialization. If equilibrium is efficient, the first chosen reporting manager truthfully satisfies the information demand of the decision maker and the decision maker accepts the report. Alger and Salanié [47] assumed that there are positive levels of Θ and an endogenous $E_{i}^{b}$. The model I developed extends these models in two ways. First, whereas Wolinsky’s [2] asymmetrical-information-advantaged supplier is able to reject the order of a report, in my model, the reporting manager cannot reject the order due to the superior-subordinate relationship in the chain of command. Furthermore, the decision maker has the option to reject a report. Second, the decision maker could overestimate the demand, which was not envisaged in the models of Wolinsky [2] and Alger and Salanié [47].

Three kinds of equilibria—efficient equilibria, specialization equilibria, and overloading equilibria—are analyzed, and the necessary and sufficient conditions for the existence of these types of equilibria are discussed.

**Definition 1:** *If the first chosen reporting manager provides a report that satisfies the decision maker’s information demand and the decision maker accepts*, *then the equilibrium is efficient*.

Let the transfer price set of the first chosen reporting manager be (E^*b*^, E′, E*). Note that competing reporting managers are present (*b*, *ε*_*γ*_, *ε*_*γ*_). It is assumed that the decision maker accepts both a small report and a large report. To prevent the reporting manager from overloading a decision maker if a minor information demand exists, the reporting manager’s benefits when recommending a small report have to exceed the benefits of reporting a large one, that is, E*−*ε*_*γ*_−Θ≤E′−*ε*_*γ*_, which can be simplified to E*−Θ≤E′.

If the reporting manager offers a small report with E′, then the decision maker knows that the information demand requests a small report due to Assumption 3. It is assumed that there are reporting managers who specialize in providing small reports or large reports. Because of uncertainty, the decision maker might reject a large report and instead asks a small report specialist instead. According to Fong [50], decision makers might be tempted to accept an information underload when the expected costs of not receiving information are higher than the costs of receiving insufficient information (information underload). Taking this into account, it is necessary for the efficient equilibrium that E′≤*b*+*ε*_*γ*_. That implies that the advantage of economies of scope cannot be used to increase the reporting manager’s transfer price. Combined with the prior condition, both inequalities imply


$$E^{*}-\varepsilon_{\gamma }-\Theta +E^{\prime }\leq E^{\prime }-\varepsilon_{\gamma }+b+\varepsilon_{\gamma },$$ (2)

which can be easily transformed to


$$\Theta \geq \varepsilon_{\Gamma }-b-\varepsilon_{\gamma }=\Theta^{e}.$$ (3)

Consequently, Θ≥Θ^*e*^ can be interpreted as a sufficient condition for the existence of an efficient equilibrium. This leads to the following proposition.

**Proposition 1:** An efficient equilibrium exists with the conditions that the decision maker accepts a report and the reporting manager provides an adequate set of information if and only if *Θ*≥*Θ*^*e*^. From the decision maker’s view, for any *Θ*≥*Θ*^*e*^, each transfer price set $\left(b-\mu (\varepsilon_{\Gamma }-\varepsilon_{\gamma }-\Theta ),\varepsilon_{\Gamma }-\Theta ,\varepsilon_{\Gamma }\right)$ is an equilibrium transfer price. The expected cost of reporting for the decision maker is $IC=b+\varepsilon_{\gamma }\mu +\varepsilon_{\Gamma }(1-\mu )$. From the reporting manager’s view, the additional benefit beyond the benefit of providing a report is zero.

Note that it is not profitable for reporting managers to deviate in their reporting transfer prices if Θ≥Θ^e^.

In the (S1 Appendix), I show that the reporting managers are not tempted to provide an inadequate (adequate) report. Therefore, E′≤*b*+*ε*_*γ*_, or equivalently, E′−*ε*_*γ*_≤*b* is a consequence of Θ≥Θ^*e*^.

In conclusion, there is an elemental trade-off between reporting incentives, which indicates that E* should be at a high level, and that E′ should be small enough due to the decision maker’s opportunism to get the report for a lower transfer price from another reporting manager. In terms of information overload, the gap between E* and E′ is the decisive factor. For the remainder of this article, that gap is defined as Ξ. That implies that there are incentives for reporting managers, which are determined by E*, that must be great enough to encourage the reporting manager to provide a report. To discourage the reporting managers from overloading the decision maker, E*−E′, or Ξ, must be small. In turn, E′ must be small too, or the decision maker would not accept the recommended kind of report (i.e., large or small report).

Consequently, if Θ is not able to sustain an efficient equilibrium, then some inefficiencies will arise. These inefficiencies may occur in specialization or overloading equilibria, which are discussed in the following.

**Definition 2:** *A specialization equilibrium is an equilibrium in which the decision maker inquires with a reporting manager who specializes on small reports and truthfully recommends a type of report in the briefing*. *If a small report is needed*, *then the decision maker accepts the report offered; otherwise the decision maker inquires with another reporting manager*, *whose offer of a large report would be accepted by the decision maker*.

The specialization equilibrium describes the possible desire of the decision maker to get a second opinion and is based on Wolinsky [2]. Even though an overloading behavior does not arise in the discussed equilibria, the decision maker could tend to hedge.

Consider the following kind of game. There are at least two reporting managers who specialize on small reports with the transfer price set (*b*, *ε*_*γ*_, *ε*_*γ*_), and at least two reporting managers who specialize on large reports with the transfer price set (*b*, *ε*_*γ*_, *ε*_Γ_). The decision maker as well as the reporting managers exhibit the behaviors described in Definition 2. In this kind of game, the reporting managers who specialize on small reports would report truthfully, but the decision maker would reject a small report if a major information demand is present. However, reporting managers who specialize on large reports overload decision makers they would accept large reports even if they only need small ones. From these reporting managers’ view, informational overloading leads to an increase in transfer prices because Θ≤*ε*_Γ_−*ε*_*γ*_, or equivalently, *ε*_*γ*_≤*ε*_Γ_−Θ. It is assumed that Θ<Θ^*e*^.

If Θ^*s*^≤Θ^*e*^ exists, it would lead to


$$b+\varepsilon_{\Gamma }>b+\varepsilon_{\gamma }\mu +\varepsilon_{\Gamma }(1-\mu ),$$ (4)

or equivalently,


$$\varepsilon_{\Gamma }>\varepsilon_{\gamma }\mu +\varepsilon_{\Gamma }(1-\mu ).$$ (5)

Under this condition, the dominating strategy of the decision maker is to inquire with a reporting manager who specializes on large reports.

**Proposition 2:** *A specialization equilibrium exists with the conditions that the reporting managers who specialize on small reports have the transfer price set* (*b*, *ε*_*γ*_, *ε*_*γ*_) *and reporting managers who specialize on large reports have the transfer price set* (*b*, *ε*_*γ*_, *ε*_Γ_), *which are equilibrium transfer prices*. *The reporting managers expect to make a zero benefit*. *Such a specialization equilibrium is characterized by* Θ∈[Θ^*s*^, Θ^*e*^].

According to the briefing costs, the condition Θ^*e*^≥Θ^*s*^ for a specialization equilibrium implies that


$$(\varepsilon_{\Gamma }-\varepsilon_{\gamma })\frac{\mu }{1-\mu (1-\mu )}>b$$ (6)

which is proved in the (S2 Appendix).

That condition requires that the costs of the inefficient briefings (an involvement of the specialization equilibrium) should not be large. It is obvious that the higher the probability that the decision maker needs a small report (represented by *μ*), the higher the expected benefit of the reporting manager from specialization. Furthermore, a wide range between the costs of the providing the reports *ε*_Γ_ and *ε*_γ_ would imply an increasing transfer price from specialization. Moreover, the economies of scope (*b*>0) leads to a situation where inquiring with another reporting manager entails an additional transfer price when a large report is needed.

Consequently, that implies that if *b* would be large and *μ* would be small, the decision maker would tend to get the large report from the first reporting manager inquired, even if that would mean information overloading. From the decision maker’s view, there is an essential trade-off between costly information overload and costly repetition of briefings. Therefore, information overload occurs as an equilibrium phenomenon. This equilibrium would not hold when the risk of overloading is small.

**Definition 3:** *An overloading equilibrium is an equilibrium in which the decision maker inquires with a reporting manager who always recommends a large report*, *and the decision maker accepts the first recommendation*.

The strategic behavior of a reporting manager *j*, who always provides a large report, has to overcome the cost of informational overloading Θ in any case in which the decision maker’s information demand is minor. The cost of providing a report is *IC*+Θ*μ*. The second term shows the overloading equilibrium’s inefficiency. Considering Propositions 1 and 2, that leads to the following.

**Proposition 3:** *There is an overloading equilibrium if and only if the conditions* Θ<Θ^*e*^ *and* Θ<Θ^*s*^ *are fulfilled*. *The reporting managers who always offer a large report have the transfer price set* (E^*b*^, E′, E*). The reporting managers do not expect to make a zero benefit. The equilibrium transfer price set is (E^*b*^, E′, E*) = (*b*−(*ε*_Γ_−*ε*_γ_−Θ)*μ*, *ε*_Γ_,*ε*_Γ_).

In the (S3 Appendix), I show that an overloading temptation could have a positive transfer price if and only if Θ≤Θ^*s*^. Obviously, this implies that in an overloading equilibrium, the transfer price of a report is high and does not depend on the true information demand of the decision maker. If the briefing costs are large enough, the decision maker might be tempted not to first inquire with a reporting manager who specializes on small reports. That is shown by *b*>(*ε*_Γ_−*ε*_γ_)*μ*, which is a necessary condition for Θ≥0. Note that even in a competitive setting, information overload can occur as an equilibrium phenomenon. Moreover, concerning information underload, there is no equilibrium in which the reporting manager provides a small report if a major information demand is present.

Consequently, after determining the necessary and sufficient conditions for the existence of three types of equilibria—efficient equilibria, specialization equilibria, and overloading equilibria—the following proposition deals with the exclusivity of these three types of equilibria. Note that it is assumed that the decision maker’s information demand must be satisfied by providing a report.

**Proposition 4**: There is either an efficient equilibrium, a specialization equilibrium, or an overloading equilibrium if the costs of briefing are positive (*b*>0).

**Fig 2:**
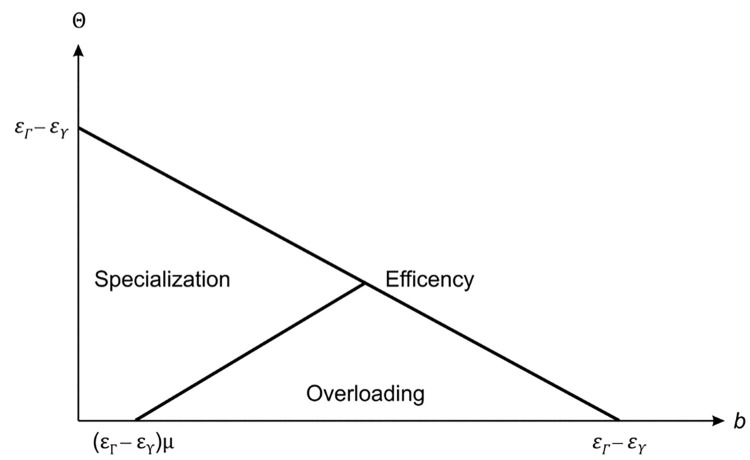
Equilibria and the interrelationship between specificity and briefing costs.

Dulleck and Kerschbamer [5] show that in the case where Θ = *ε*_Γ_−*ε*_γ_, equilibria are efficient. In the same case, Alger and Salanié [47] consider that a positive *b* leads to the existence of one of the three equilibria types. In the converse case where Θ = 0, Wolinsky [2] implies that even recommendations by an overloading reporting manager are costless and thus, efficient (see also S4 Appendix). A graphical representation of the relations between the efficient equilibria, specialization equilibria, and overloading equilibria is shown in Fig 2. Note that the summary of the equilibria is shown in Table 1.

**Table 1:** Equilibrium relation of Θ.

	Relation of *Θ*	Equilibrium Transfer price Set	Solutions
**Efficient equilibrium**	*Θ*≥*Θ*^*e*^	$\left(b-\mu (\varepsilon_{\Gamma }-\varepsilon_{\gamma }-\Theta ),\varepsilon_{\Gamma }-\Theta ,\varepsilon_{\Gamma }\right)$	${\Theta^{e}=\varepsilon }_{\Gamma }-b-\varepsilon_{\gamma }$
**Specialization equilibrium**	*Θ*^*s*^≤*Θ*<*Θ*^*e*^	(*b*, *ε*_*γ*_, *ε*_*Γ*_)	$\Theta^{s}=(b-\mu (\varepsilon_{\Gamma }-\varepsilon_{\gamma }))\frac{1-\mu }{\mu^{2}}$
**Overloading equilibrium**	*Θ*<*Θ*^*s*^,*Θ*<*Θ*^*e*^	$\left(b-(\varepsilon_{\Gamma }-\varepsilon_{\gamma }-\Theta )\mu ,\varepsilon_{\Gamma },\varepsilon_{\Gamma }\right)$	

This table shows the summary of the three different equilibria.

To determine the area of overloading, we determine the point of intersection of the two curves. That point of intersection is $(\frac{\mu (\varepsilon_{\Gamma }-\varepsilon_{\gamma })}{\mu^{2}-\mu +1}\left|\varepsilon_{\Gamma }-\varepsilon_{\gamma }-\frac{\mu (\varepsilon_{\Gamma }-\varepsilon_{\gamma })}{\mu^{2}-\mu +1}\right)$.

From Fig 2, it is obvious that the decision maker’s overloading threat is influenced by two factors: the gap between *ε*_Γ_ and *ε*_γ_ and the probability of a minor information demand *μ*. If that gap is small, the slope of Θ^e^ is flat, which implies that the area of the overloading equilibria would be small. In turn, that means that the greater the gap, the greater the slope, and the greater the area of overload. In terms of probability *μ*, the level of *μ* only affects the x-intercept, but not the slope of Θ^s^. If *μ* is at a high level, then the area of overload is small. Consequently, the area of overload is the integral $\frac{{\left(\varepsilon_{\Gamma }-\varepsilon_{\gamma }\right)}^{2}{\left(1-\mu \right)}^{3}}{2(1-\mu +\mu^{2})}$. This underlines the importance of the two factors. The higher the gap between *ε*_Γ_ and *ε*_γ_ and the lower the probability of a minor information demand *μ*, the higher is the information overload.

## Effects on corporate performance

Research has found that information overload reduces decision making performance [13, 14, 35]. If corporate performance is linked with the decision making performance of the decision maker, then an SID overload will reduce corporate performance. As seen in the inverted-U curve, the relation between information load and decision making performance can be defined as


$$L(x)=-x^{2}+2\vartheta x+\omega ,$$ (7)

where *x* is the set of information, *ϑ* is the set of information that marks the highest level of decision making performance, and *ω* is the set of information the decision maker already knows without inquiring with the reporting manager. Several experimental studies found that *ϑ* is optimal at a value between five and eight [13, 14, 44, 51–57]. The partial derivative of *L*(*x*) is $\frac{\partial L}{\partial x}=-2x+2\vartheta$ > 0 and diminishing marginal information is similarly taken to correspond to $\frac{\partial^{2}L}{\partial x^{2}}$ < 0.

Concerning the three types of equilibria, the information load is optimal in an efficiency equilibrium, which implies that *x*_*s*_ = *x*_*d*_. The corporate performance is optimal ceteris paribus. In a specialization equilibrium, the decision maker might exhibit a major (minor) information demand and receive a large (small) report. In that kind of equilibrium, *x*_*s*_ = *x*_*d*_. Only in an overloading equilibrium does the reporting manager provide a large report to the decision maker who has a minor information demand. In that case, *x*_*s*_>*x*_*d*_.

**Fig 3:**
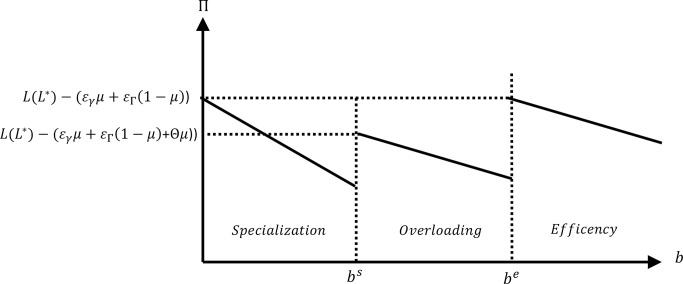
Corporate performance.

In this model, corporate performance is measured by decision making performance minus the expected cost of reporting, which depends on the decision maker’s information demand. Therefore, corporate performance is defined as $\Pi^{e}=L(L^{*})-IC=L(L^{*})-(b+\varepsilon_{\gamma }\mu +\varepsilon_{\Gamma }(1-\mu ))$ for efficient equilibria, $\Pi^{s}=L(L^{*})-(IC+(1-\mu )b)=L(L^{*})-({(\varepsilon }_{\gamma }-\varepsilon_{\Gamma }(1-\frac{1}{\mu }))\mu +(2-\mu )b)$ for specialization equilibria, and $\Pi^{o}=L(L^{*})-(IC+\Theta \mu )=L(L^{*})-(b+(\varepsilon_{\gamma }+(1-\frac{1}{\mu })\varepsilon_{\Gamma }+\Theta )\mu )$ for overloading equilibria. The different levels of corporate performance are shown in Fig 3.

## Discussion

If Assumption 1 (competition) would be relaxed, there would be no competing reporting managers. That implies that the mark-up above a small report effort is not driven down by other reporting managers who offer small reports at a lower transfer price level. In that case, there might exist other equilibria where no reporting manager offers a specialist transfer price set. Hence, there could exist other equilibria without information overload by reporting managers. That leads to the following proposition.

**Proposition 5:** Without reporting managers who offer (*b*, *ε*_*γ*_, *ε*_*γ*_), there exists an efficient equilibrium if and only if the conditions Θ≥Θ^*e*^ *and* Θ≤Θ^*s*^ *are fulfilled*.

To illustrate, suppose that reporting managers are competing to win the decision maker’s favor. They are able to react quickly if one of them tries to undercut the others. That situation may change if the decision maker discovers the nature of the information demand, and could use a software tool to remedy the lack of information. That do-it-yourself alternative is depicted by Assumptions 1 and 3. Therefore, efficient equilibria disappear for sufficiently small overload costs. Consequently, the competition threat by other reporting managers or by self-information could be a decisive force behind information overloading.

If Assumption 4 (absence of overconfidence) and Assumption 3 are relaxed, the decision maker’s tendency to demand a large report increases. Therefore, it would be easier for (specialized) reporting managers to recommend a large report, even if the information demand is minor. In place of *μ* there would be *M*, defined as *M* = *μ*−*μ*_*O*_, where *μ*_*O*_ is the level caused by overconfidence (see S5 Appendix). This implies that *M* is systematically biased by overconfidence, which has been assumed and confirmed by previous studies [13, 14, 17–20].

## Conclusions

Information overload can be the result of SID. The information-supplying reporting manager could overload the decision maker deliberately due to information asymmetry, uncertainty about the decision maker’s information demand, and inefficient incentive structures. If there are reporting managers who specialize in different kind of reports, the decision maker’s risk of being overloaded increases.

The starting point of this study is the fact that the intracorporate information providing process can be seen as expert market with information asymmetry. Assuming a limited information processing capacity of the decision maker, information overload is the result of informational overconsumption caused by the information-supplying reporting manager. This study links the studies of Schroder et al. [29], Chervany and Dickson [21], and Chewning and Harrell [13] with the SID problem, as discussed in Wolinsky [2] and Diamond [46].

The strategic content of information tempts reporting managers to overload the decision maker for the purpose of increasing their reporting transfer price and to discourage the decision maker from getting the information from another reporting manager. Although the decision maker knows a part of the information demand, information overload involves the cost of using unnecessary inputs. From the corporate view, information overload reduces decision making performance and therefore decreases performance. The model of this study contributes to information overload theory in several aspects.

First, information overload occurs as an SID of information, even if other competing reporting managers exist. This finding may offer a structural basis for applied research on reporting manager-induced information demand. Second, the provided model may explain why briefing efforts are often greater than briefing transfer prices. As in most models with switching costs, reporting managers want to draw the decision maker’s attention in the first period. This analysis assumes that reporting mark-ups may pervade in expert markets, either as an instrument to overload, or as a direct result of overloading. Finally, the mere threat of information overload may reduce corporate performance, even in the absence of an overloading equilibrium, by tempting the decision maker to seek a costly double consulting (specialization equilibrium).

My analysis demonstrates that information overload can occur due to uncertainty and opportunism of both the decision maker and reporting managers. I see two immediate avenues for future research. First, there is a need for evidence that systematically distinguishes the effects of deliberate strategic information overload and negligent information overload. In particular, reporting managers could use information overload to influence decisions purposefully. Secondly, there is still a lack of evidence concerning the effects of information overload in SID situations. Experimental evidence would allow a true out-of-sample test of the model developed here.

## Supporting information

S1 Appendix Proof of Proposition 1. (PDF)

S2 Appendix Proof of Proposition 2. (PDF)

S3 Appendix Proof of Proposition 3. (PDF)

S4 Appendix Proof of Proposition 4. (PDF)

S5 Appendix Proof of Proposition 5. (PDF)

## References

[1] Darby M, Karni E. Free Competition and the Optimal Amount of Fraud. Journal of Law and Economics [Internet] 1973;16(1):67–88. Available from: https://econpapers.repec.org/article/ucpjlawec/v_3a16_3ay_3a1973_3ai_3a1_3ap_3a67-88.htm.

[2] Wolinsky A. Competition in a Market for Informed Experts’ Services. The RAND Journal of Economics 1993;24(3):380–298.

[3] Evans RG. Supplier-Induced Demand: Some Empirical Evidence and Implications. In: Perlman M, editor. The Economics of Health and Medical Care: Proceedings of a Conference held by the International Economic Association at Tokyo. International Economic Association Series. London, s.l.: Palgrave Macmillan UK; 1974. p. 162–73.

[4] Labelle R, Stoddart G, Rice T. A re-examination of the meaning and importance of supplier-induced demand. Journal of Health Economics 1994;13(3):347–68. doi: 10.1016/0167-6296(94)90036-1 10138860

[5] Dulleck U, Kerschbamer R. On Doctors, Mechanics, and Computer Specialists: The Economics of Credence Goods. Journal of Economic Literature 2006;44(1):5–42.

[6] Bevan G. Has There Been Supplier-Induced Demand for Legal Aid? Civil Justice Quarterly 1996;15:98–114.

[7] Tata C. In the Interests of Clients or Commerce? Legal Aid, Supply, Demand, and ’Ethical Indeterminacy’ in Criminal Defence Work. Journal of Law and Society 2007;34(4):489–519.

[8] Blanchard WA. Relevance of Information and Accuracy of Interpersonal Prediction: A Methodological Note. Psychological Reports 1966;18(2):379–82.

[9] Gross BM. The Managing of Organizations: The Administrative Struggle. New York: Free Press of Glencoe; 1968.

[10] Naylor J, Clark P. Human Inference Behavior as a Function of Validity and Magnitude of Sign. Organizational Behavior and Human Performance 1968;4:378–98.

[11] Porat A, Haas J. Information Effects on Decision Making. Behavorial Science 1969;41:98–104.

[12] Nystedt L. Consensus Among Judges as a Function of Set of information. Educational and Psychological Measurement 1974;34(1):91–101.

[13] Chewning EG, Harrell AM. The effect of information load on decision makers’ cue utilization levels and decision quality in a financial distress decision task. Accounting, Organizations and Society 1990;15(6):527–42.

[14] Tuttle B, Burton F. The effects of a modest incentive on information overload in an investment analysis task. Accounting, Organizations and Society 1999;24(8):673–87.

[15] Roetzel PG. Information overload in the information age: a review of the literature from business administration, business psychology, and related disciplines with a bibliometric approach and framework development. BuR—Business Research 2019;12(2):479–522.

[16] Feldman MS, March JG. Information in Organizations as Signal and Symbol, Vol 26; 1981.

[17] Fertakis JP. On Communication, Understanding, and Relevance in Accounting Reporting. The Accounting Review [Internet] 1969;44(4):680–91. Available from: http://www.jstor.org/stable/243669.

[18] Abdel-Khalik AR. The Effect of Aggregating Accounting Reports on the Quality of the Lending Decision: An Empirical Investigation. Journal of Accounting Research 1973;11:104–38.

[19] Shields MD. Some effects on information load on search patterns used to analyze performance reports. Accounting, Organizations and Society 1980;5(4):429–42.

[20] Snowball D. Information Load and Accounting Reports: Too Much, Too Little or Just Right? Cost and Management 1979;5:22–8.

[21] Chervany NL, Dickson GW. An Experimental Evaluation of Information Overload in a Production Environment. Management Science 1974;20(10):1335–44.

[22] Bawden D. Information and digital literacies: a review of concepts. Journal of Documentation 2001;57(2):218–59.

[23] Schneider SC. Information overload: Causes and consequences*. HSM 1987;7(2):143–53.

[24] Galbraith JR. Organization Design: An Information Processing View. Interfaces [Internet] 1974;4(3):28–36. Available from: http://www.jstor.org/stable/25059090.

[25] Schick AG, Gordon LA, Haka S. Information overload: A temporal approach. Accounting, Organizations and Society 1990;15(3):199–220.

[26] Tushman ML, Nadler DA. Information Processing as an Integrating Concept in Organizational Design. The Academy of Management Review 1978;3(3):613–24.

[27] O’Reilly CA. Individuals and Information Overload in Organizations: Is More Necessarily Better? The Academy of Management Journal 1980;23(4):684–96.

[28] Miller GA. The magical number seven, plus or minus two: Some limits on our capacity for processing information. Psychological Review 1956;63(2):81–97. 13310704

[29] Schroder HM, Driver MJ, Streufert S. Human Information Processing: Individuals and Groups Functioning in Complex Social Situations. New York: Holt, Rinehart & Winston of Canada; 1967.

[30] Milord JT, Perry RP. A Methodological Study of Overloadx. Journal of General Psychology 1977;97(1):131–7. doi: 10.1080/00221309.1977.9918509 28137202

[31] Simon HA. Information processing models of cognition. Annual Review of Psychology 1979;30:363–96. doi: 10.1146/annurev.ps.30.020179.002051 18331186

[32] Revsine L. Data Expansion and Conceptual Structure. The Accounting Review [Internet] 1970;45(4):704–11. Available from: http://www.jstor.org/stable/244209.

[33] Iselin ER. The effects of information load and information diversity on decision quality in a structured decision task. Accounting, Organizations and Society 1988;13(2):147–64.

[34] Simnett R. The effect of information selection, information processing and task complexity on predictive accuracy of auditors. Accounting, Organizations and Society 1996;21(7–8):699–719.

[35] Swain MR, Haka SF. Main Articles—Effects of Information Load on Capital Budgeting Decisions. Behavioral research in accounting 2000;12:171–99.

[36] Achoff RL. Management Misinformation Systems. Management Science 1967;14(4):147–56.

[37] Jacoby J. Perspectives on Information Overload. Journal of Consumer Research [Internet] 1984;10(4):432–5. Available from: http://www.jstor.org/stable/2488912.

[38] Malhotra NK. Reflections on the Information Overload Paradigm in Consumer Decision Making. Journal of Consumer Research 1984;10(4):436–40.

[39] Keller KL, Staelin R. Effects of Quality and Quantity of Information on Decision Effectiveness. Journal of Consumer Research 1987;14(2):200–13.

[40] Simon HA, Newell A. Human problem solving: The state of the theory in 1970. American Psychologist 1971;26(2):145–59.

[41] Driver MJ, Streufert S. Integrative Complexity: An Approach to Individuals and Groups as Information-Processing Systems. Administrative Science Quarterly 1969;14(2):272.

[42] Driver MJ, Mock TJ. Human Information Processing, Decision Style Theory, and Accounting Information Systems. The Accounting Review [Internet] 1975;50(3):490–508. Available from: http://www.jstor.org/stable/245007.

[43] Driver MJ, Brousseau KR, Hunsaker PL. The Dynamic Decision Maker: Five Decision Styles for Executive and Business Success. New York: Harper & Row; 1990.

[44] Eppler MJ, Mengis J. The Concept of Information Overload: A Review of Literature from Organization Science, Accounting, Marketing, MIS, and Related Disciplines. The Information Society 2004;20(5):325–44.

[45] Farley PJ. Theories of the price and quantity of physician services. A synthesis and critique. Journal of Health Economics 1986;5(4):315–33. doi: 10.1016/0167-6296(86)90007-x 10282332

[46] Diamond PA. A model of price adjustment. Journal of Economic Theory 1971;3(2):156–68.

[47] Alger I, Salanié F. A Theory of Fraud and Overtreatment in Experts Markets. Journal of Economics and Management Strategy 2006;15(4):853–81.

[48] Ronen J, Yaari V. Demand for the truth in principal–agent relationships. Rev Acc Stud 2007;12(1):125–53.

[49] Nan N. A principal‐agent model for incentive design in knowledge sharing. Journal of Knowledge Management 2008;12(3):101–13.

[50] Fong Y. When Do Experts Cheat and Whom Do They Target? The RAND Journal of Economics [Internet] 2005;36(1):113–30. Available from: http://www.jstor.org/stable/1593757.

[51] Ashton RH. An Experimental Study of Internal Control Judgements. Journal of Accounting Research 1974;12(1):143–57.

[52] Joyce EJ. Judgment in Audit Program Planning, Studies on Human Information Processing in Accounting. Journal of Accounting Research 1976;14:29–60.

[53] Hofstedt TR, Hughes GD. An Experimental Study of the Judgment Element in Disclosure Decisions. The Accounting Review [Internet] 1977;52(2):379–95. Available from: http://www.jstor.org/stable/245416.

[54] Savich RS. The Use of Accounting Information in Decision Making. The Accounting Review [Internet] 1977;52(3):642–52. Available from: http://www.jstor.org/stable/246084.

[55] McGhee W, Shields MD, Birnberg JG. The Effects of Personality on a Subject’s Information Processing. The Accounting Review [Internet] 1978;53(3):681–97. Available from: http://www.jstor.org/stable/245675.

[56] Schultz JJ, Gustavson SG. Actuaries’ Perceptions of Variables Affecting the Independent Auditor’s Legal Liability. The Accounting Review [Internet] 1978;53(3):626–41. Available from: http://www.jstor.org/stable/245671.

[57] Danos P, Imhoff EA. Auditor Review of Financial Forecasts: An Analysis of Factors Affecting Reasonableness Judgments. The Accounting Review [Internet] 1982;57(1):39–54. Available from: http://www.jstor.org/stable/246738.

