# Correction: The role of supplier-induced demand on the occurrence of information overload in managerial reporting environments

**DOI:** 10.1371/journal.pone.0331337

**Published:** 2025-08-28

**Authors:** The PLOS One Staff

## Notice of Republication

This article was republished on August 11, 2025, to correct an error in the citation. The correct citation is: Rötzel PG (2024) The role of supplier-induced demand on the occurrence of information overload in managerial reporting environments. PLoS One 19(7): e0307671. https://doi.org/10.1371/journal.pone.0307671x
