# Supplementary material for: The role of supplier-induced demand on the occurrence of information overload in managerial reporting environments
Source: PLoS One. 2024 Jul 25;19(7):e0307671. doi: 10.1371/journal.pone.0307671 (PMC11271863; doi:10.1371/journal.pone.0307671)
Supplement: S1 Appendix — (PDF) [file pone.0307671.s001.pdf]

S1 Appendix. Proof of Proposition 1.

First, if $\Theta \geq \Theta^e$ and all reporting managers choose the same transfer price set $(\mathrm{E}, \mathrm{E}', \mathrm{E}^*) = (b - \mu(\varepsilon_\Gamma - \varepsilon_\gamma - \Theta), \varepsilon_\Gamma - \Theta, \varepsilon_\Gamma)$, then an efficient equilibrium exists. In this efficient equilibrium, the reporting manager's transfer price for the briefing is $\mathrm{E}^b \geq 0$. Furthermore, the assumption of observable characteristic inputs for large reports ($\Theta \in [0, \varepsilon_\Gamma - \varepsilon_\gamma]$) would imply that $\varepsilon_\gamma < \mathrm{E}'$. There might be three different kinds of reporting managers (large report specialist, small report specialist, all-arounder); (1) if the reporting managers could provide only large reports, then $\Theta = \varepsilon_\Gamma - \varepsilon_\gamma$ and $\mathrm{E}^* = \varepsilon_\Gamma$. The reporting manager will gain the transfer price E^* for providing a large report. A pricing of small reporting is not necessary as only large reports are provided. If the decision maker forces the reporting manager to name a transfer price, then the reporting manager would name E^*. Due to Assumption 2, the decision maker will accept the report offered. In cases of minor information demand, the decision maker rejects the offer. For this reason, this kind of reporting manager gets no benefits by satisfying minor information demand. However, by naming E^*, the reporting manager might get $\mathrm{E}^* - \varepsilon_\gamma - \Theta$, for it is assumed that the decision maker would accept the offer. Hence, $\mathrm{E}^* - \varepsilon_\gamma - \Theta < 0$, due to the conditions $\mathrm{E}^* \geq \varepsilon_\Gamma$ and $\Theta \leq \varepsilon_\Gamma - \varepsilon_\gamma$. This leads to $\mathrm{E}^* = \varepsilon_\Gamma$ and $\Theta = \varepsilon_\Gamma - \varepsilon_\gamma$. (2) In turn, the reporting manager could only provide small reports and never large reports. Conversely to the prior case, $\mathrm{E}^* < \varepsilon_\Gamma$. According to Assumption 2, if the decision maker first inquires with this reporting manager, the reporting manager can only offer E' or the decision maker rejects the offer. (3) A reporting manager who provides both small reports and large reports could always offer a large report. Without Assumption 2, the decision maker would pay $\mathrm{E}^b + \mathrm{E}^*$. However, the decision maker could have first inquired with a small report specialist (competition condition), so $b + \varepsilon_\gamma \mu + (\mathrm{E}^b + \mathrm{E}^*)(1 - \mu) \geq (\mathrm{E}^b + \mathrm{E}^*)$. Consequently, $\mathrm{E}' \leq b + \varepsilon_\gamma$, or equivalently, $\mathrm{E}' - \varepsilon_\gamma \leq b$ is a consequence of $\Theta \geq \Theta^e$. This completes the proof.
