# Supplementary material for: The role of supplier-induced demand on the occurrence of information overload in managerial reporting environments
Source: PLoS One. 2024 Jul 25;19(7):e0307671. doi: 10.1371/journal.pone.0307671 (PMC11271863; doi:10.1371/journal.pone.0307671)
Supplement: S2 Appendix — (PDF) [file pone.0307671.s002.pdf]

S2 Appendix. Proof of Proposition 2.

Consider that there is a reporting manager who provides small reports and large reports, and always recommends a large report to the decision maker for a transfer price of

$$\mathrm{E}^b + \mathrm{E}^* \in [IC + \Theta\mu, b\frac{1}{\mu} + \varepsilon_y], \tag{8}$$

which implies that $\Theta = \Theta^s$, in terms of a specialization equilibrium. It is assumed that the briefing costs are positive ($b > 0$), because otherwise ($b = 0$) the reporting manager's knowledge of the information demand of the decision maker would be obtained at no cost. Furthermore, the existence of economies of scope implies $b > 0$. Consequently, the game would be simplified to a Bertrand game in this special case.

To prove the existence of a specialization equilibrium, denote the transfer price sets (E_α^b, E'_α, E_α^*) and (E^b, E', E^*) for the groups of specialized reporting managers for small reports (α) and large reports. At equilibrium, the reporting managers who specialize on large reports would provide large reports ($\mathrm{E}^* \geq \varepsilon_\Gamma$) and the transfer prices are nonnegative ($\mathrm{E}^b + \mathrm{E}^* \geq \varepsilon_\Gamma + b$). If the decision maker has a major information demand and inquires with a reporting manager who specializes on small reports, then the decision maker would reject the offer of a small report, which leads to $\mathrm{E}_\alpha^* > \mathrm{E}^b + \mathrm{E}^*$. In a specialization equilibrium, the benefits are zero. Concerning Assumption 2, reporting managers who specialize on small reports make no benefit. In terms of reporting managers who specialize on large reports, their transfer price is

$$(\mathrm{E}^b + \mathrm{E}^* - (\varepsilon_\Gamma + b))(1 - \mu) \geq 0 \tag{9}$$

Finally, the possibility of deviation of the reporting managers who specialize on large reports must be taken into account. Concerning the proof of Proposition 1 (efficient equilibrium), there is a reporting manager i who tries to attract the decision maker with μ by

choosing the transfer price set (E_i^b, E_i', E_i^*). There are three possible combinations of report-size recommendation and the decision maker's acceptance to discuss. Firstly, suppose the decision maker would accept a small report as well as a large report. In this case, $\Theta \geq \Theta^e$ and $b > 0$ must hold. In turn, $b = 0$ would imply that the deviation of the reporting manager would lead to a zero benefit. Secondly, the decision maker would turn down any recommendation. That implies that $\mathrm{E}_i^b < 0$, which would attract the decision maker. In this case, the reporting manager would be faced with a negative transfer price. Thirdly, the decision maker is confident that a large report is absolutely necessary. The reporting manager would not recommend a small report when a large report can be easily provided. In the case of providing a large report, the transfer price must be

$$\mathrm{E}_i^b + \mathrm{E}_i^* \geq IC + \Theta\mu \tag{10}$$

If $\mathrm{E}_i^b + \mathrm{E}_i^* \geq \varepsilon_\Gamma + b$, the decision maker would not inquire with the reporting manager i. In turn, if $\mathrm{E}_i^b + \mathrm{E}_i^* < \varepsilon_\Gamma + b$, the decision maker who first inquired with another reporting manager who specializes on small reports would inquire with the reporting manager i. This implies that the level of $\varepsilon_\Gamma + b$ should not be too low, because otherwise the reporting manager i would be faced with a negative benefit by offering that transfer price. Therefore, the reporting manager i would get a benefit from reporting if and only if the decision maker inquires promptly. That implies that

$$b + \varepsilon_\Upsilon\mu + (IC + \Theta\mu)(1 - \mu) \geq (IC + \Theta\mu), \tag{11a}$$

or equivalently,

$$\left(b - \mu(\varepsilon_\Gamma - \varepsilon_\Upsilon)\right)\frac{1-\mu}{\mu^2} > \Theta \tag{11b}$$

Consequently,

$$\Theta^s = \left(b - \mu(\varepsilon_\Gamma - \varepsilon_\gamma)\right)\frac{1-\mu}{\mu^2} \tag{12}$$

This completes the proof.
