# Supplementary material for: The role of supplier-induced demand on the occurrence of information overload in managerial reporting environments
Source: PLoS One. 2024 Jul 25;19(7):e0307671. doi: 10.1371/journal.pone.0307671 (PMC11271863; doi:10.1371/journal.pone.0307671)
Supplement: S3 Appendix — (PDF) [file pone.0307671.s003.pdf]

S3 Appendix. Proof of Proposition 3.

The key problem with this kind of equilibrium is that information overload could occur due to overloading by a reporting manager. The reason is that there are reporting managers who could provide both small reports and large reports. If these reporting managers would truthfully recommend a small report if needed, they would not maximize their benefit. Moreover, it might be important for the reporting manager to bind the decision maker and to prevent a defection due to the presence of switching cost. In this case, the decision maker might never learn what kind of report is really needed in order to make a decision. From the decision maker's view, the overloading causes additional costs of $\Theta\mu$.

Concerning the proof of Proposition 1, there is a reporting manager j who tries to attract the decision maker with beliefs μ by choosing the transfer price set (E_j, E_j', E_j^*). There are four possible combinations of report-size recommendation and the decision maker's acceptance to discuss. Firstly, suppose the decision maker would accept a small report as well as a large report. Then $\Theta \geq \Theta^e$ and $b > 0$, because there is another reporting manager providing a small report. In turn, $b = 0$ is not discussed because of the assumption of economies of scope as described in the prior proofs. Secondly, the decision maker would turn down any recommendation. That implies that $\mathrm{E}_j < 0$ would attract the decision maker. However, in this case, the reporting manager j would be faced with a negative transfer price. Thirdly, the decision maker is confident that a large report is absolutely necessary. The reporting manager j would not recommend a small report a large report can be easily provided. In turn, the reporting manager has to be concerned that the decision maker would turn down the recommendation of a small report. That implies that $\mathrm{E}_j^* - \varepsilon_\gamma - \Theta \leq 0$, and in combination with $\mathrm{E}_j^* \geq \varepsilon_\Gamma$, that leads to $(\varepsilon_\Gamma - \varepsilon_\gamma - \Theta) \leq 0$. Hence, $\Theta \geq \Theta^e$ is required, which is not an equilibrium. Lastly, suppose that the decision maker only accepts recommendations for small reports. The proof of Proposition 2 implies that such a transfer price set would tempt the decision maker to accept

the first inquiry. Therefore, that temptation could have a positive transfer price if and only if $\Theta \leq \Theta^s$. This completes the proof.

The core issue with the equilibrium under discussion is the potential for information overload, which can occur due to varying report sizes provided by a reporting manager. This is problematic because some managers may choose between delivering small or large reports depending on their incentives. Ideally, a manager would recommend a smaller report when it suffices, but this might not align with their interest in maximizing benefits. Additionally, the manager might aim to commit the decision maker to a course of action, particularly when switching costs are significant, thereby preventing defection. Consequently, the decision maker might fail to discern the necessary report size to make an informed decision, resulting in excessive information processing costs denoted by Θμ.

In the detailed scenario of Proposition 1, consider reporting manager j who is targeting decision maker with beliefs μ by setting a transfer price set (E_j, E_j', E_j^*). I analyze four possible scenarios:

1. Acceptance of Any Report Size: If the decision maker is willing to accept both small and large reports, then $\Theta \geq \Theta^e$ and $b > 0$, indicating that another manager provides a more competitively priced small report. The scenario where $b = 0$ is excluded by the assumption of economies of scope, which was established in previous proofs.
2. Rejection of All Recommendations: If the decision maker rejects all recommendations, then setting $\mathrm{E}_j < 0$ would be necessary to attract the decision maker. However, this leads to a negative transfer price for manager j, which is not feasible.
3. Requirement of a Large Report: When the decision maker is convinced of the necessity of a large report, manager j would not recommend a small report since providing a large report is straightforward. However, if reporting manager j recommends a small report, it risks rejection. This leads to the condition $\mathrm{E}_j^* - \varepsilon_\gamma - \Theta \leq 0$, and in combination with

$E_j^* \geq \varepsilon_\Gamma$, simplifying to $(\varepsilon_\Gamma - \varepsilon_\gamma - \Theta) \leq 0$. Thus, a threshold Θ^e is required, indicating a non-equilibrium condition.

4. Exclusive Acceptance of Small Reports: According to the proof of Proposition 2, a transfer price set favoring only small reports would be tempting if it enables acceptance at the first inquiry. This is only feasible if $\Theta \leq \Theta^s$, aligning with the equilibrium conditions.

This structured analysis clarifies the conditions under which different report sizes and transfer pricing strategies influence the decision maker's choices, addressing the risks of information overload and misalignment of reporting incentives.
