# Supplementary material for: The role of supplier-induced demand on the occurrence of information overload in managerial reporting environments
Source: PLoS One. 2024 Jul 25;19(7):e0307671. doi: 10.1371/journal.pone.0307671 (PMC11271863; doi:10.1371/journal.pone.0307671)
Supplement: S4 Appendix — (PDF) [file pone.0307671.s004.pdf]

S4 Appendix. Proof of Proposition 4.

If there are no costs of briefing ($b = 0$), then the decision maker would always discover the true state (i.e., whether a small report or a large report is needed). Moreover, there would not be any economies of scope between the briefing and the providing of the report for the reporting manager. However, if $b > 0$ then there are economies of scope such that the briefing is dominated by a specialization equilibrium. In that case, the reporting manager who specializes on small reports reveals the true state and might provide the small report if the information demand of the decision maker is minor.
