# Supplementary material for: The role of supplier-induced demand on the occurrence of information overload in managerial reporting environments
Source: PLoS One. 2024 Jul 25;19(7):e0307671. doi: 10.1371/journal.pone.0307671 (PMC11271863; doi:10.1371/journal.pone.0307671)
Supplement: S5 Appendix — (PDF) [file pone.0307671.s005.pdf]

S5 Appendix. Proof of Proposition 5.

Assume that all reporting managers would set $(\mathrm{E}, \mathrm{E}', \mathrm{E}^*) = (b - \mu(\varepsilon_\Gamma - \varepsilon_\gamma - \Theta), \varepsilon_\Gamma - \Theta, \varepsilon_\Gamma)$, which marks an efficient equilibrium. Furthermore, suppose all reporting managers truthfully recommend the report type. This implies that $\Theta \geq \Theta^e$, which was shown in the proof of Proposition 1. In turn, if $\Theta < \Theta^e$, then reporting managers who specialize in small reports would offer $\mathrm{E}' \geq b + \varepsilon_\gamma$. If there is a possibility that a reporting manager could deviate to such a specialist, then the non-deviating reporting manager could expect that the decision maker would reject the offer of a small report. Due to that expectation, the non-deviating reporting managers would always recommend large reports, which leads to an overloading equilibrium (Proof of Proposition 3). If $\Theta \leq \Theta^s$, then the decision maker would inquire with a non-deviating specialist even though that leads to expected costs of $\Theta\mu$. That implies that deviation is not advantageous for reporting managers. This completes the proof.
